# MALDI-TOF MS in a Medical Mycology Laboratory: On Stage and Backstage

**DOI:** 10.3390/microorganisms9061283

**Published:** 2021-06-12

**Authors:** Marie-Gladys Robert, Muriel Cornet, Aurélie Hennebique, Tahinamandranto Rasamoelina, Yvan Caspar, Léa Pondérand, Marie Bidart, Harmonie Durand, Marvin Jacquet, Cécile Garnaud, Danièle Maubon

**Affiliations:** 1 Centre National de la Recherche Scientifique, Centre Hospitalo-Universitaire Grenoble Alpes, Institut National de la Santé et de la Recherche Médicale, Institut Pour l’Avancée des Biosciences, University Grenoble Alpes, 38000 Grenoble, France; MRobert2@chu-grenoble.fr; 2 Translational Innovation in Medicine and Complexity, Centre National de la Recherche Scientifique, Centre Hospitalo-Universitaire Grenoble Alpes, Grenoble Alpes University, 38000 Grenoble, France; MCornet@chu-grenoble.fr (M.C.); AHennebique@chu-grenoble.fr (A.H.); MJacquet6@chu-grenoble.fr (M.J.); CGarnaud@chu-grenoble.fr (C.G.); 3 Charles Mérieux Infectiology Center, Campus Universitaire d’Ankatso, Antananarivo 101, Madagascar; ninamandranto@yahoo.fr; 4 Institut de Biologie Structurale, Commissariat à l’Énergie Atomique et aux Énergies Alternatives, Centre National de la Recherche Scientifique, Centre Hospitalo-Universitaire Grenoble Alpes, University Grenoble Alpes, 38000 Grenoble, France; YCaspar@chu-grenoble.fr (Y.C.); lponderand@chu-grenoble.fr (L.P.); 5 Clinatec Departement, University Grenoble Alpes, Centre Hospitalo-Universitaire Grenoble Alpes, 38000 Grenoble, France; Mbidart@chu-grenoble.fr (M.B.); durand.harmonie@gmail.com (H.D.)

**Keywords:** MALDI-TOF MS, medical mycology, diagnosis, microorganism identification, fungi, antifungal susceptibility testing

## Abstract

The implementation of MALDI-TOF MS in medical microbiology laboratories has revolutionized practices and significantly reduced turnaround times of identification processes. However, although bacteriology quickly benefited from the contributions of this technique, adjustments were necessary to accommodate the specific characteristics of fungi. MALDI-TOF MS is now an indispensable tool in clinical mycology laboratories, both for the identification of yeasts and filamentous fungi, and other innovative uses are gradually emerging. Based on the practical experience of our medical mycology laboratory, this review will present the current uses of MALDI-TOF MS and the adaptations we implemented, to allow their practical execution in a daily routine. We will also introduce some less mainstream applications, like those for fungemia, or even still under development, as is the case for the determination of sensitivity to antifungal agents or typing methods.

## 1. Introduction

Although the concept has been proposed for more than 40 years for microbiology applications, MALDI-TOF MS has only started to be used extensively over the 10 last years for microorganisms’ identification in clinical laboratories [1,2,3,4]. The highly specific protein spectra generated by this technology is like a barcode (also named MSP by Bruker, for Main Spectra Profiles) which allows the identification of microorganisms up to the species level within a few minutes. The accuracy of identification relies on previously created databases (also known as ‘libraries’) that include thousands of reference spectra obtained from strains strictly identified by DNA sequencing. More recently MALDI-TOF MS commercial assays have also been successfully used to evidence antibiotic resistant phenotypes in bacteria, or to accelerate the identification of microorganisms directly from positive blood cultures.

Medical mycology is a discipline that deals with human pathogenic microscopic fungi, which are basically divided into two categories: yeasts and filamentous fungi. Fungal infections can be very common and harmless, such as mucocutaneous candidiasis or dermatophytosis, but also rare and extremely pathogenic particularly in immunocompromised patients, as for invasive aspergillosis, cryptococcosis or mucormycosis. Additionally, although resistant phenotypes are described in fungi of medical importance (FMI), they are still much less frequently reported than for bacteria. These considerations of low incidence rates in infection and resistance compared to the bacterial world, can explain why, when MALDI-TOF MS was implemented in diagnostic laboratories, mycologists sometimes felt that their discipline was second in line, behind bacteriology, in terms of innovative development and commercial strategies. With this in mind, and also because MALDI-TOF MS is an easy-to-use technology, many mycologists initially developed their own tools or adapted existing protocols to better meet their everyday and specific expectations. In this review, we will present the undeniable benefits of MALDI-TOF MS but also the limits of this technology in the specific context of FMI identification. Additionally, we will share our experiences and drawbacks, and present the solutions we choose to implement in our laboratory to get the best from mass spectrometry.

## 2. The Place of MALDI-TOF MS in Yeast Identification

### 2.1. Routine Use

Due to its ease of use, rapidity, accuracy and low reagents costs, MALDI-TOF MS is now routinely used as a first-line technique for the identification of common yeasts. In addition, as identification processes are very similar for both bacteria and yeasts, and based on the same systems and reagents, MALDI-TOF MS is a central part of microbiology laboratory automation. CLSI therefore established guidelines for microbiological identification by MALDI-TOF MS (https://clsi.org/standards/products/microbiology/documents/m58/, accessed on 3 May 2021).

MALDI-TOF MS allows species identification directly from a colony forming unit (CFU). As for bacteria, the colony can be directly transferred to the target plate the matrix is added. However, a pre-extraction step is sometimes required for yeast identification. Two protocols of extraction are classically described ((Table 1): (i) On-plate (or rapid) protein extraction by adding formic acid or (ii) Off-plate ethanol/formic acid (or long/full) extraction [5,6]. As the latter is time-consuming, the on-plate extraction protocol is the most commonly used.

Both handling time and turnaround time of MALDI-TOF MS are limited. Indeed, as opposed to most of the phenotypic methods currently available for yeast identification, MALDI-TOF MS does not require a subculture step (except if the colony is not pure) and so speeds up the identification. In addition, it only requires a small amount of colony and can therefore be performed from an early culture. It was shown that identification was obtained on average 1.45 days earlier than with current methods [7]. Using direct deposit, time-to-result from colony is less than 15 min.

In addition to its rapidity, the other main advantage of MALDI-TOF MS is its accuracy. It improved yeast identification compared to common phenotypic techniques by discriminating very close species or subspecies. For instance, depending on the database, MALDI-TOF MS makes it possible to distinguish species among the *Candida* complexes such as *C. parapsilosis* complex (*C. parapsilosis stricto sensu*, *C. metapsilosis* and *C. orthopsilosis*), *C. glabrata* complex (*C. glabrata s.s*, *C. bracarensis*, *C. nivariensis*) or the *C. haemulonis* complex (*C. haemulonis s.s.*, *C. pseudohaemulonii* and the emerging species *C. auris*). The species within and the *C. neoformans* and the *C. gattii* complexes can also be distinguished according to the recently changed taxonomy [5,8,9].

Both databases of the two main MALDI-TOF MS systems currently available, MALDI Biotyper (Bruker Daltonics GmbH, Bremen, Germany) and VITEK MS/MS Plus (bioMérieux, Marcy l’Etoile, France), include common yeasts isolated in clinical microbiological laboratories. While there may be some differences in nomenclature (anamorph/teleomorph) or species between these two databases, their performances for common yeast identification appear similar with high rates of identification, from 83 to 99%, for common yeasts (see review Patel 2019 for more details [5]). The Autof MS 1000 system (Autof Acquirer database V2.0.18, Autobio Diagnostics Co., Ltd, Zhengzhou, China) also appears suitable for identifying common yeasts [10].

Comparing performances of the different MALDI-TOF MS systems is rather challenging because of the multiplicity of extraction procedures and reference techniques, as well as the evolution of databases. Indeed, identification rates depend on the process used for identification: off-plate extraction yielded higher identification rates than on-plate extraction, which itself gave higher identification rates than direct deposit. Several authors suggested that the latter was therefore inappropriate for yeast identification when using the same recommendations as for bacteria (i.e., for Bruker: score ≥2.0 and polished-steel target) because of low quality spectra [6,11,12]. However, lowering the threshold from 2.0 to 1.7 (Table 1) helped increase identification rates after direct deposit, without changing the misidentification rates thus making direct transfer suitable for routine use (personal experience and [6,9]). In addition, the use of biotargets or ground-steel targets rather than polished-steel ones also increased the identification rates after direct transfer. Finally, culture conditions including type of media may influence the performance of MALDI-TOF MS for yeast identification [6].

### 2.2. Limits and Solutions

MALDI-TOF MS presents only a few limitations for common yeast identification. Misidentifications and no identifications are rare. No identification cases mainly result from the absence of the given yeast species in the database, from the poor quality of the spectra or from undetected polymicrobial colonies. In the latter case, duplicate, repeat testing, or re-isolation of pure CFU often lead to a correct identification.

Identification is highly dependent on the quality of the databases (number of species, number of spectra per species, etc.). The large majority of common yeast species are included in these databases, but as they are constantly evolving, regular updates are necessary. Apart from the commercial IVD and RUO databases, user-developed on-line databases are freely available (Microbenet developed by the CDC (https://microbenet.cdc.gov; accessed on 3 May 2021) or the Mass Spectrometry Identification platform (MSI, available online at https://msi.happy-dev.fr/ (accessed on 3 May 2021)), or can be developed for a specific project when commercial databases are challenged, as can be the case for rare yeasts (Table 1). For instance, such a database was recently developed to improve the identification of South-American strains of *C. auris* which were not included in the MALDI-TOF MS Bruker Biotyper OC 3.1.66 database [13]. MALDI-TOF MS identification of *Cryptococcus neoformans* subspecies can also be challenging and requires pre-extraction and development of such user-developed bases as well as peak analysis process [5,9,14].

In addition, databases are most often developed using culture conditions and long protein extraction procedures that can differ from the ones routinely used in microbiological laboratories, which explains why identification performances are not always perfect. Spectra obtained after direct deposit can present important noise whereas those obtained using rapid formic acid extraction seem to be more compatible with these databases [6].

Several other factors can influence the quality of the spectra, and notably the quality of the deposit on the target. Even though this may seem trivial, it requires training to transfer the right amount of colony on the plate. Adequate training may also improve the performances of yeast identification after direct deposit [5].

Regular maintenance of the MALDI-TOF MS system, and especially the laser calibration, is also crucial for the quality of the obtained spectra [15].

If reagent costs are limited (restricted to internal control and matrix solutions, plates and devices for the deposit of colony and reagents), the MALDI-TOF system itself remains expensive for laboratories of low-income countries [16]. In addition, most databases are commercial ones with updates which are usually included in the maintenance contract.

## 3. The Place of MALDI-TOF MS in Common Filamentous Fungi Identification

### 3.1. Routine Use

Morphological identification, which is based on the examination of the macroscopic and microscopic characteristics of fungal cultures, is the conventional method for identifying filamentous fungi in medical mycology laboratories. However, this method relies on some expertise, can be subjective, and requires sufficient incubation time to ensure the development of fruiting bodies. Moreover, it can be insufficiently discriminating in certain cases. Indeed, some species may have similar morphological characteristics and be difficult to differentiate. This is the case of *Rasamsonia* spp. which are frequently misidentified as *Penicillium* spp. or *Paecylomyces* spp. [17]. The example of cryptic species also perfectly illustrates the limitations of morphological identification. These genetically different but morphologically identical species can have significantly different antifungal susceptibility profiles, making their reliable identification at the species level a key issue for the introduction of appropriate treatment [18,19]. Sequencing of specific DNA regions can be used for accurate identification, but this method is cumbersome, expensive and difficult to integrate in a routine clinical laboratory setting.

MALDI-TOF MS was first proposed for the identification of filamentous fungi in 2000 by Welham et al. [20], but it is only in the last ten years that it has been able to be implemented routinely thanks to the development of adapted protocols and specific libraries, which was initiated within the medical mycology laboratories themselves [21].

Current guidelines for culture conditions and sample preparation protocols vary between authors, MALDI-TOF MS platform manufacturers or library suppliers. The simplest protocol consists of a direct deposit of a small part of the colony grown on solid media and the addition of formic acid on the same principle as the on-plate protocol mentioned above for yeast, but the quality of the spectra can be improved by using longer protocols requiring subculture in liquid media or involving a bead-beating step and/or an ethanol-formic acid-acetonitrile extraction [22,23]. For example, in view of identification on the MALDI Biotyper^®^ (Bruker Daltonics GmbH, Bremen, Germany). Bruker recommends direct extraction from the solid medium in the first instance (Table 1). If this rapid procedure fails, an off-plate extraction protocol requiring an additional ten minutes is proposed from the same solid medium. As a last resort, a culture in liquid medium can be carried out, but this protocol, although perfectly in line with the generation mode of the Bruker library reference spectra, extends the identification time by at least 24 h.

Specific commercially available libraries are now proposed by platform manufacturers, allowing simplified real-time identification of the most common filamentous FMI.

### 3.2. Limits

The introduction of MALDI-TOF MS has undoubtedly facilitated the identification of filamentous fungi in clinical microbiology laboratories, but these extremely diverse eukaryotic organisms remain difficult to identify by this method due to several limiting factors. First, from a technical point of view, molds can be difficult to sample from the solid media on which they are conventionally grown due to their high adherence. Correct sampling without agar which could contaminate the spectra, can be time-consuming and require trained personnel. The on-plate protocol, more suitable for routine use, may be insufficient to obtain quality spectra, and the use of longer but more standardized protocols may be necessary.

Secondly, although manufacturers’ libraries are regularly updated to include more species and cover the variations encountered within a single species, facing the great diversity that can be encountered in human pathology, they still suffer from being incomplete. For instance, the Bruker filamentous Fungi database (V3) covers 189 species, and the VITEK MS database only 79 fungal species [24]. Hence, if the most common molds are generally well covered, things become more complicated when it comes to rarer ones or cryptic species. The identification thresholds proposed by manufacturers may also not be adapted to this specific use [25,26].

Finally, a recent multicenter study identified the spectra acquisition parameters—e.g., the peak evaluation parameters or the signal noise threshold—and the instrument maintenance as factors that can limit identification performance [27].

### 3.3. Solutions

Adaptation strategies have had to be considered in order to overcome these limiting factors. Concerning the difficulties of collecting fungal colonies, we recently evaluated the Id Fungi Plates (IDFP, Quincieux, Lyon, France), a new solid media covered by a porous membrane specifically developed to facilitate the sampling of molds and the identification by mass spectrometry (Table 1). Compared to Sabouraud media, IDFP allowed an easier and therefore faster harvest (92.4% vs. 9.1%), and more identification without the need for off-plate protocols [28]. Other teams also concluded that this media improves the identification of filamentous fungi [29,30]. We therefore routinely use these new media for the subculture of clinical isolates not suggestive of *Aspergillus*, for which collection and identification is more challenging.

Regarding the identification thresholds, when using a MALDI Biotyper (Bruker Daltonics GmbH, Bremen, Germany), several authors suggest that, as for yeast, reducing the species cutoff proposed by Bruker from 2 to 1.7 increases the number of correct identifications with no significant impact on the misidentification rate [25,26] (Table 1).

The deficiencies of commercial libraries have compelled laboratories to develop their own databases. Some of these libraries are commercially or freely available, such as the NIH Mold Database [23], Microbenet developed by the CDC which provides reference spectra for more than 400 FMI, such as rare dimorphic fungi, or the MSI platform (Table 1). The latter is currently the most comprehensive database covering more than 1000 fungal species (including dermatophytes), and clearly improves the routine use of MALDI-TOF MS for mold identification. Using this library, Normand et al. reached 87.35% of correct identifications at the species level on a panel of 501 clinical isolates, vs. 60.24% or 25.7% using the Bruker database and cutoffs of 1.7 and 2.0, respectively [31]. Based on our experience of the routine identifications of clinical filamentous fungi, we compared the Bruker and the online MSI databases using 265 spectra (Bruker MBT System). We also found that our Bruker database (V3, 9888 entries, 1340 fungi) lacks reference spectra for specific genera such as *Galactomyces*, *Corynascus*, *Paecilomyces*, *Dicyma* and *Acrodontium*, making them either unidentifiable or misidentified (unpublished data). In our hands, MSI outperformed Bruker with 76% of identification to the species level versus 54%, respectively. In addition, Bruker’s database offers limited discrimination among the *Aspergillus* genus. Notably, in the *Nigri* section (*A. tubingensis* and *A. welwitschiae* misidentified as *A. niger*), the *Fumigati* section (*A. thermomutatus* misidentified as *A. lentulus*) and the *Nidulantes* section (*A. spinulosporus* misidentified as *A. nidulans*) for which antifungal susceptibility differs greatly [19,32,33]. Overall, our results confirmed a previous study showing that the MSI database provided more accurate identifications than Bruker and that the systematic combination of the two databases significantly improves the rate of correct identifications for filamentous fungi [34]. However, a recent evaluation of the MSI platform on 245 Aspergillus isolates covering 56 cryptic species and 13 sections reported that although 99.6% of identifications were correct at the section level, only 66.1% of isolates were correctly identified at the species level [35]. Moreover, Nabet et al. recently showed that almost 40% of mold clinical isolates from a medical mycology laboratory in a tropical area (French Guyana) could not be identified by MALDI-TOF MS using the Bruker filamentous fungi database and the MSI platform [36]. Therefore, there is still room for improvement, and libraries must be constantly updated.

## 4. The Place of MALDI-TOF MS in Rare Filamentous Fungi Identification

### 4.1. Routine Use

As detailed in the previous paragraph, an optimal identification of common filamentous fungi with MALDI-TOF MS still needs some adaptation regarding the culture media or the database used (Table 1). When it comes to rare filamentous fungi, protocols are the same as for common ones, but the main limit is the rarity or the absence of reference spectra in the commercial databases. Nevertheless, unexpected species are sometimes evidenced in a specific clinical context. As an example, due to the fact that the species *Schizophyllum commune* was present in the Bruker library in 2016, we and others could identify this fungus as a common but previously poorly described species involved in sinusitis or bronchopulmonary diseases [37,38]. *S. commune* usually do not sporulate under classical culture conditions which makes morphological identification almost impossible. Rare previously published case reports identified *S. commune* with a sequencing approach [39], which is not of classical routine use in medical mycology. In this context, one could say that MALDI-TOF MS has been the most efficient tool for both mycologists and clinicians to identify and manage this new clinical entity.

### 4.2. Limits

As previously mentioned, the main limit in the identification of uncommon filamentous fungi is the rarity of reference spectra in commercial databases. In fact, a non-negligible number of spectra exist in the Bruker library, but a high number of those are from fungal species that are not of medical importance, but more designed for plant health approach, or food industry [40]. Of note, thanks to the regular updates of commercial databases, this drawback is less disabling than before, but still not entirely satisfactory.

### 4.3. Solutions

Again, some solutions need to be found to optimize identification of rare FMI. We were confronted with this issue in 2018, when we failed to identify a fungal like infection of the cornea in a young patient [41]. All attempts to identify this mycelium obtained from the corneal biopsy on the basis of microscopic morphology or by MALDI-TOF MS failed. Although the spectra were nice and clean, they could not be attributed to any of the microorganisms present at that time in the Bruker Taxonomy (7815 MSP), Bruker Filamentous Fungi (364 MSP), NIH mold (365 profiles) or MSI fungal (1026 species) databases. Confirmation of the species was finally achieved by sequencing and revealed the fungal-like oomycete named *Pythium insidiosum*. Treatment was consequently changed and the patient’s eye was saved, albeit just in time. Here, a non-negligible amount of time was lost because no reference spectra for *P. insidiosum* were present in the various databases commonly used at that time for microbial identification [40]. Therefore, after confirmation by sequencing, we created our own reference MSP on the Microflex mass-spectrometer, according to Bruker’s MSP creation protocol (v1.1). This reference spectrum was not only added to our local database but also shared on the new version of the MSI database, to facilitate future diagnosis of pythiosis for both our institute and other healthcare centers [41].

Creating home-made ‘libraries’ is time-consuming and requires technical skills, and yet it remains the best solution to implement new rare species of medical importance (Table 1). This strategy was recently used in 2016 in our laboratory in the context of a large epidemiological study of sporotrichosis and chromoblastomycosis in Madagascar [42], which aimed at facilitating the diagnosis of these endemic dermo-invasive mycosis. For that purpose, and because reference spectra were extremely rare in the commercial databases at that time (2 MSPs in the Bruker database and 1 profile in the NIH database for *Sporothrix* sp., and none for the different agents of chromoblastomycosis), we generated 20 and 11 reference spectra from reference or previously sequenced strains for *Sporothrix schenkii*, and chromoblastomycosis causative agents (i.e., *C. carrionii*, *F. nubica*, *F. pedrosoi*, and *F. monophora*), respectively [43,44]. These homemade libraries now allow us to accurately and rapidly identify these agents, without the need for DNA sequencing.

## 5. The Place of MALDI-TOF MS in Positive Blood Culture Identification

### 5.1. Routine Use

Rapid and appropriate first-line antifungal therapy has been shown to improve outcomes in patients with fungemia, but identification is often delayed by the need to subculture positive blood cultures [45]. MALDI-TOF MS has been used for over 10 years to identify species directly from positive blood cultures [46,47]. In bacteriology, another method to identify microorganisms from blood culture is to perform MALDI-TOF MS on short-incubation microcolonies, but because some yeasts are slow-growing this strategy has not proved to be very efficient [48,49].

As the sample also contains red and white blood cells, the protocols must start with either lysis or filtration steps in order to remove human proteins. In-house protocols based on the lytic agent saponin have been rapidly and successfully described for bacterial identification [50], but only a few assays have been developed for yeast positive blood cultures [51]. Three commercial assays are now on the market: the MBT-Sepsityper^®^ kit (Bruker Daltonics GmbH, Bremen, Germany), the VITEK MS blood culture kit (bioMerieux, Marcy l’Etoile, France), and the rapid BACpro^®^ II kit (Nittobo Medical Co., Tokyo, Japan) [52] (Table 1).

In 2014 we evaluated the MBT Sepsityper^®^ assay, which gave good results for the characterization of bacteria and yeasts with short time-to-results (TTR) of less than 30 min [53]. This kit was then optimized with the development of the ‘Rapid Sepsityper^®^’ protocol which reduces the number of centrifugation steps, and thus allows identification of bacteria and yeasts in 10 min. In parallel, Bruker developed a specific MBT-Sepsityper module for spectra analysis, designed to increase identification performance. In 2019, we evaluated this identification strategy (Rapid Sepsityper and MBT-Sepsityper module) from positive blood cultures [54]. Concerning yeast identification, standard Sepsityper protocol, based on ethanol and formic acid extraction appeared to be more effective than Rapid-Sepsityper. Importantly, the use of specific MBT-Sepsityper module increased the identification rates of yeasts by 38% [54]. Of note, whatever the protocol, identification rates were always higher with the addition of formic acid [54].

### 5.2. Limits

Although the MBT-Sepsityper and the Rapid Sepsityper provide interesting results in terms of identification accuracy and rapidity, a major drawback is their lack of automation. Even with the Rapid Sepsityper (which is, as previously mentioned, not optimal for yeast identification), when a high number of positive blood cultures have to be handled, it still requires a lot of technical steps, especially when compared to the fully automated identification system based on PCR or microarray [55,56]. Another limiting issue is the price of the test, to be added to the cost of the interpretation module MBT-Sepsityper. Even though it is far below the cost of fully automated PCR identification methods considering the additional staff required to perform the test, it becomes a non-negligible issue in large healthcare centers dealing with a high number of positive blood cultures.

At last, today, and contrary to PCR solutions, no MALDI-TOF MS commercial solutions exist for the direct identification of pathogens directly applied on patients’ blood samples [56,57,58].

### 5.3. Solutions

In order to limit costs, in 2015, we tested and compared different in-house protocols for the direct identification of the yeast species commonly isolated from blood cultures by MALDI-TOF MS [53]. We compared the results obtained with our home-made assays to those obtained with the Sepsityper kit. We found that blood culture lysis with 1.8% SDS was globally superior to the Sepsityper kit, particularly for yeasts isolated from Mycosis IC/F medium (Bactec system, Bruker Daltonics GmbH, Bremen, Germany) and for non-albicans *Candida* species (Table 1). The two protocols had similar durations (30 min), but the SDS 1.8% protocol was far cheaper than the Sepsityper protocol (by a factor of 220 [0.025 compared to 5.5 euros per test]) [53].

We then successfully tested this home-made SDS 1.8% protocol using the same rapid protocol as the one developed later by Bruker in the Rapid Sepsityper [59]. Results were comparable to the ones obtained in the following study using Rapid Sepsityper [54,59], confirming that home-made SDS 1.8% protocol is suitable for both bacteria and yeast identifications.

Bruker has also developed a specific software for the analysis and interpretation of spectra obtained directly from blood cultures, which takes into account that spectra obtained from pathogens isolated from blood cultures are less ‘clean’ than the ones obtained from CFUs on a Petri dish. We also evaluated the potential benefit of this specific MBT Sepsityper RUO software compared to the standard MBT Compass-IVD software for spectrum analysis from positive blood culture to reliably identify bacteria and yeasts to the species level without modifying cut-offs provided by the manufacturers. Considering yeast identification, the MBT Sepsityper-RUO module provided significantly higher percentages of reliable identifications to the species level than the MBT Compass-IVD module. However, only the algorithms used for interpretation differ between these 2 software: the analyzed spectra are exactly the same. Before the commercialization of this specific module, we and others had shown that lowering the thresholds and defining new criteria for interpretation were suitable for routine microorganisms identification at the species level when dealing with blood cultures, for instance a score between <2 but >1.7 and a species proposed at least four times in a row in the identification process [53,60,61].

In brief, for a given spectra obtained from a blood culture, the MBT Sepsityper RUO software could give a higher identification score than a standard software, but our personal opinion is that lowering the identification scores with well-defined and validated limits is equivalent (Table 1).

Another interesting and efficient approach would be the ability of MALDI-TOF MS to directly detect specific pathogens markers from the patient blood, thus saving precious time for clinical management.

In 2016, Mery et al. demonstrated that MALDI-TOF MS was able to detect a disaccharide compound in the serum of patients suffering from invasive fungal infection (invasive candidiasis, aspergillosis and mucormycosis) [62]. In their study, when positively detected, this pan-fungal new biomarker could be detected earlier than the classical clinical diagnostic. Although its sensitivity was around 83% per patient (and 62% on a single serum), it appeared to be an interesting, easily detected and cheap biomarker to use in conjunction with already existing tests in the context of invasive fungal infection suspicion (Beta-D-glucans, Galactommann, or mannan detection). This proof-of-concept is still under validation on larger patient cohorts, but appears a promising and easy-to-use tool for invasive fungal detection (Table 1).

**Table 1:** Description of the different MALDI-TOF MS protocols existing in the field of medical mycology, and of their possible alternatives.

Application for		Recommendation/Protocols	Possible Alternatives for FMI
Yeast identification	Common Yeast identification	Formic acid Off plate extraction On plate extraction Regular maintenance and laser calibration	Direct deposit on biotargets or ground steel targets Score threshold adaptation
	Rare yeast identification	Check presence in commercial databases	Confront spectra to other databases (MSI, NHI, MicrobNet)
		Update databases	Create homemade reference libraries
Mold identification	Common mold identification	Long protocol extraction, bead beating, subculture in liquid media, EtOH-AF *	On plate extraction Off plate extraction Score threshold adaptation Specific culture or subculture media (IDFP^®^) Use of MSI, NHI, MicrobNet for accurate identification of cryptic species
	Rare mold identification	Check presence in commercial databases	Confront spectra to other databases (MSI, NHI, MicrobNet) Homemade reference libraries
Fungemia	On positive blood culture	Commercial kit MBT-Sepsityper^®^ (Bruker Daltonics, Bremen, Germany) Rapid Sepsityper^®^ (Bruker Daltonics, Bremen, Germany) VITEK MS ^®^ blood culture kit (bioMerieux, Marcy l’Etoile, France rapid BACpro^®^ II kit (Nittobo Medical Co., Tokyo, Japan) Sepsityper RUO software (Bruker Daltonics, Bremen, Germany)	In house protocols based on 1.8% SDS solution Score threshold adaptation
	On patient blood	No commercial solution	Disaccharide detection [62] Proof of concept
Antifungal susceptibility testing	On Strains	Incubation with ATF	Spectra profile changes analysis Growth inhibition analysis (MBT Astra) Strain typing
		Specific R or S peaks direct detection	Proof of concept on *C. glabrata* Further development is needed

* EtOH-AF: ethanol formic-acid acetonitrile extraction; FMI: fungi of medical importance; ATF: Antifungal; R: resistant phénotype; S sensitive phenotype.

## 6. The Place of MALDI-TOF MS in Antifungal Susceptibility Testing

### 6.1. Routine Use

There is no currently commercial solution for a routine use of antifungal susceptibility testing (AFST) by MALDI-TOF MS. Efforts for the development of methods to use MALDI-TOF MS for AFST initially relied on the hypothesis that protein composition would change after drug exposition (Table 1). The proof-of-concept was successfully achieved on *C. albicans* with varying concentrations of fluconazole over 15 h of incubation [47]. Then, simplifications were made leading to the analysis of a matrix of 3 concentrations and a reduction of duration of the experiment to 3 h [63,64]. Triazoles were tested with *C. albicans*, *C. tropicalis* and *C. glabrata* [65,66] and echinocandins with the *C. parapsilosis* complex [67]. For *C. glabrata* and anidulafungin 85% of agreement was obtained in 3 h and increased to 97% in 12 h, suggesting that incubation time is critical [68]. Recently, *C. auris* echinocandin resistance was detected after a three-hour incubation of three concentrations of anidulafungin [69]. AFST has also been used to determine the susceptibility profile to voriconazole of *Aspergillus* spp. [70]. MBT-ASTRA also use the MALDI-TOF technology but it is quite different as it provides a semi-quantitative analysis of the strain growth inhibition (Table 1). It is based on analyzing the intensity of the peaks and areas under curve (AUC) displayed by the spectrum that can be correlated to the growth [71,72]. MBT-ASTRA sensitivity was higher than that of the spectrum changes method (96% vs. 85.3%), while the specificity was similar for both approaches (93.2% vs. 94.2%, respectively). Interestingly, the pooled sensitivity and specificity of the methods with a turnaround times below 8 h were higher (91.4% and 96.1%, respectively) than those of the method with a turnaround time above 8 h (86.3% and 87.5%, respectively) [73]. Thus, with overall sensitivity and specificity of 91% and 95%, respectively, the MALDI-TOF MS-based AFST has shown encouraging results and may be considered as a new method for the rapid detection of resistance in Fungi.

**Figure 1:**
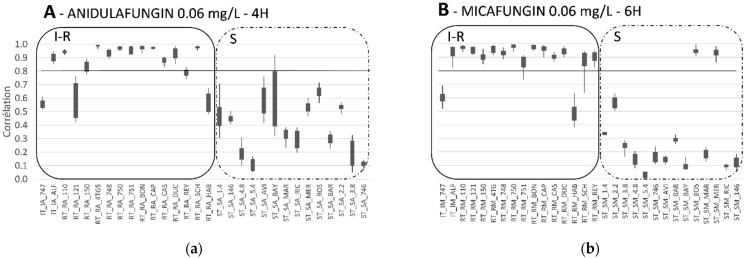
Antifungal susceptibility testing with MALDI-TOF MS: example with echinocandin resistant *Candida glabrata* strains. (**a**) Panel A: Strains were incubated 4 h with 0.06 mg/L of anidulafungin. (**b**) Panel B: Strains were incubated 6 h with 0.06 mg/L of micafungin. S-I-R-S: sensitive- intermediate or resistant strains according to the EUCAST clinical breakpoints (https://eucast.org/astoffungi/clinicalbreakpointsforantifungals/ accessed on 3 May 2021). Each strain was tested in three different experiments, in triplicates.

In 2016, we also developed and tested an in house MALDI-TOF MS protocol specifically dedicated to the detection of echinocandin resistance in *Candida glabrata* (unpublished data). Fourteen *C. glabrata* echinocandin resistant strains, and 14 *C. glabrata* sensitive strains were submitted to 4 h incubation with or without anidulafungin (0.06 mg/L—corresponding to the SIR clinical breakpoint for this species) and to 6 h incubation with or without micafungin (0.06 mg/L). A correlation index between the two conditions (with or without anidulafungin) was established. A correlation index close to 1, indicates that whatever the presence or the absence of anidulafungin, the strain remains unchanged, thus suggesting anidulafungin resistance. For sensitive strains, spectrum profile was altered in the presence of anidulafungin or micafungin, leading to a correlation index <0.8 between the two experimental conditions. Using this protocol, and a 0.8 threshold, the detection of a resistant phenotype was achieved properly in 81.25% and 87.5% of cases, whereas a sensitive phenotype was detected for 100% and 85.7% for anidulafungin and micafungin, respectively (Figure 1).

### 6.2. Limits

Despite these promising results, especially the short time to results, and also despite many clinical laboratories already being equipped with a MALDI-TOF MS system, the MALDI-TOF AFST based methods are not matured enough to be implemented routinely. This can be due to the technically time-consuming methods that remain challenging regarding sample preparation. Therefore, they would gain in interest if these initial steps were fully automated. In addition, these methods are still heterogeneous and clearly need standardization.

### 6.3. Solutions

A future perspective is to determine the susceptibility of a strain by detecting specific peaks of either sensitive or resistant strains directly on the raw spectra (Table 1), without any incubation with the drugs, as is already the case for bacteria [74]. Determining the susceptibility profile with this approach seems possible since the association between *C. glabrata* clusters determined by MALDI-TOF and fluconazole resistance has been significantly demonstrated (Table 1). However, as some clusters comprised either susceptible and resistance strains mixed together, the accuracy of the method appears insufficient for immediate clinical use [75].

Overall, these AFST based on MALDI-TOF still need an extensive clinical validation using a more homogenous methodology where a larger number of isolates from a range of species and antifungal drugs will be necessary.

## 7. Conclusions and Perspectives

**Figure 2:**
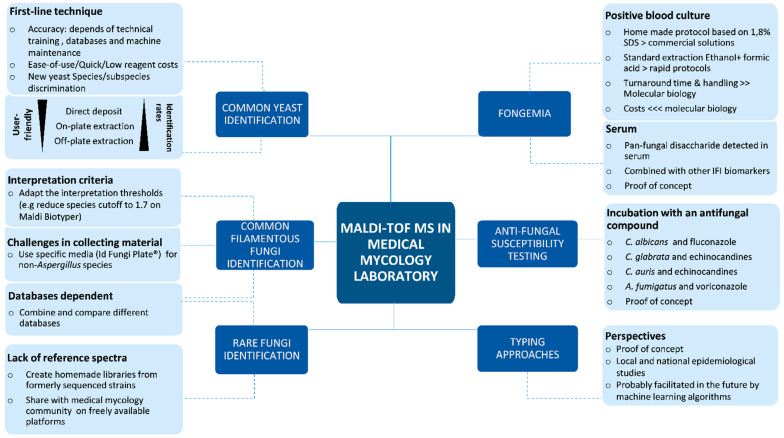
Mind map resuming the present and possible future applications, limits and solutions of MALDI-TOF MS in the field of routine use in a medical mycology laboratory.

MALDI-TOF MS has clearly become one of the most essential tools in medical mycology laboratories. Its benefits on patient management and public health have been recently evaluated [76]. Its flexibility and ease of use have made it possible to adapt or develop improved methods to better deal with the specificities of *Fungi*. During recent years, we have experienced several of these protocols, commercial or in-house, in order to tailor MALDI-TOF MS to our everyday needs (Table 1). Figure 2 summarizes the different applications and possible strategies for an optimal use of this technology in our mycology laboratory. Like others, we created in-house libraries for specific projects or rare species identification. We also combine the use of commercial databases and the MSI database for our everyday mold identification. In addition, we use the specific IDFP medium for the identification of non-Aspergillus molds. Finally, in some particular cases, we accept lower identification scores than recommended. These strategies have significantly enhanced our turnaround time and our level of accuracy for the identification of yeasts and molds. Other free databases exist such as MicrobeNet developed by the CDC and it would be interesting to evaluate its added value in our everyday identification process. More exciting applications of MALDI-TOF MS such as typing strains methods have been successfully applied by other laboratories and would be very useful for local or national epidemiological studies [77,78]. Innovative and new applications based on MALDI-TOF will probably continue to appear in the future, as illustrated by the recent demonstration of its usefulness to identify also macrofungi species and its potential applications in food safety [79]. The development of machine learning algorithms should help to transform already existing proof-of-concepts in easy and user friendly tools [80,81]. Mycology laboratories must be prepared to meet these future challenges and to continuously adapt to get the best of MALDI-TOF MS for their discipline and their patients.

## Data Availability

Data available on request due to restrictions e.g., privacy or ethical. The data presented in this study are available on request from the corresponding author. The data are not publicly available due to the fact that data were not published at the time of the experiments.
